# Socio-economic inequality in anthropometric failure among children aged under 5 years in India: evidence from the Comprehensive National Nutrition Survey 2016–18

**DOI:** 10.1186/s12939-021-01512-4

**Published:** 2021-07-30

**Authors:** Akash Porwal, Rajib Acharya, Sana Ashraf, Praween Agarwal, Sowmya Ramesh, Nizamuddin Khan, Avina Sarna, Robert Johnston

**Affiliations:** 1 Population Council, Zone 5A, Ground Floor India Habitat Centre, Lodi Road, New Delhi, Delhi 110003 India; 2 Senior Monitoring, Evaluation and Learning Expert, IPE Global Limited, New Delhi, India; 3 Nutrition Specialist, UNICEF, UNICEF House, 73 Lodi Estate, New Delhi, India

**Keywords:** Child undernutrition, CIAF, Inequality, CNNS, India

## Abstract

**Background:**

Conventional indicators used to access the nutritional status of children tend to underestimate the overall undernutrition in the presence of multiple anthropometric failures. Further, factors contributing to the rich-poor gap in the composite index of anthropometric failure (CIAF) have not been explored. This study aims to estimate the prevalence of CIAF and quantify the contribution of factors that explain the rich-poor gap in CIAF.

**Methods:**

The present study used data of 38,060 children under the age of five years and their biological mothers, drawn from the nationally representative Comprehensive National Nutrition Survey of children and adolescents aged 0–19 years in India. The CIAF outcome variable in this study provide an overall prevalence of undernutrition, with six mutually exclusive anthropometric measurements of height-for-age, height-for-weight, and weight-for-age, calculated using the World Health Organization (WHO) Multicenter Growth Reference Study. Multivariate regression and decomposition analysis were used to examine the association between covariates with CIAF and to estimate the contribution of different covariates in the existing rich-poor gap.

**Results:**

An overall CIAF prevalence of 48.2% among children aged aged under 5 years of age was found in this study. 6.0% children had all three forms of anthropometric failures. The odds of CIAF were more likely among children belonging to poorest households (AOR: 2.41, 95% CI: 2.12–2.75) and those residing in urban area (AOR: 1.06, 95% CI 1.00–1.11). Children of underweight mothers and those with high parity were at higher risk of CIAF (AOR: 1.51, 95% CI: 1.42–1.61) and (AOR: 1.15, 95% CI: 1.08–1.22), respectively. Children of mother exposed to mass media were at lower risk of CIAF (AOR: 0.87, 95% CI: 0.81–0.93).

**Conclusion:**

This study estimated a composite index to assess the overall anthropometric failure, which also provides a broader understanding of the extent and pattern of undernutrition among children. Findings show that maternal covariates contribute the most to the rich-poor gap. As well, the findings suggest that intervention programs with a targeted approach are crucial to reach the most vulnerable groups and to reduce the overall burden of undernutrition.

**Supplementary Information:**

The online version contains supplementary material available at 10.1186/s12939-021-01512-4.

## Background

Worldwide, 1 in 3 children under the age of 5 years are malnourished [1]. Undernutrition in the form of stunting or wasting affected almost 200 million children below the age of 5 years globally in 2018 [1]. Moreover, it is considered to play a major role in the premature deaths of millions of children in developing countries [2]. Those who survive are rendered vulnerable to infections and diseases, devastating the lives of hundreds of millions of children [3, 4]. The complexity of undernutrition in developing countries is amplified due to its proximate association with poverty and inequality. Evidence of socio-economic differences in maternal and child morbidity and mortality rates is well documented [5–13]. Studies have identified poverty as the leading cause of malnutrition in developing countries that leads to poor nutritional status, intergenerationally and prevent social improvement and equity [5, 8].

To evaluate the nutritional status, three conventional anthropometric indices of stunting (low height-for-age), wasting (low weight-for-height) and underweight (low weight-for-age) were calculated [9, 14]. According to the World Health Organization (WHO), these anthropometric indices reflect distinct biological processes and are necessary for determining appropriate nutritional interventions [14]. Previous studies in Asian countries have found a concurrent relationship between stunting and wasting when compared to a reference population [15]. Underweight children may experience stunting and/or wasting and some of them may experience all three forms of anthropometric failures [16]. None of the three conventional nutritional indicators can estimate the true overall burden of undernutrition in children under the age of 5 years.

Subsequently, an aggregated single anthropometric measure providing an overall estimate of under-nourished children has been proposed and known as the Composite Index of Anthropometric Failure (CIAF) [9, 17]. The original model [17] consisted of six sub-groups of anthropometric failure (Groups A–F); A: No failure, B: Only wasting, C: Wasting and underweight, D: Stunting, wasting and underweight, E: Stunting and underweight, F: Stunting only. Further, Nandy [9] stressed on an additional sub-group, Group Y, as Underweight only. The CIAF included those children experiencing stunting, underweight, wasting, and multiple failures (Groups B–Y) and excluded those children who did not exhibit any anthropometric failure (Group A). The combination of wasted and stunted was not included in the CIAF classification as it was physically impossible for a child to simultaneously experience stunting and wasting and not be underweight [9].

In India, the national prevalence of stunting and wasting in children under the age of 5 years is 37.0 and 20.8% [18], respectively, which is greater than the average of stunting and wasting from developing countries. Few recent studies confirm the existence of socio-economic inequality in prevalence of undernutrition at district level in India [19] and among the urban population [20]. However, previous studies have documented the socio-economic gap in child undernutrition based on conventional indicators of undernutrition [7, 10, 17, 19, 20]. Whereas, only few studies have documented the socio-economic gap considering CIAF as an outcome of interest, but are limited to specific geography [21]. This study utilizes data from Comprehensive National Nutrition survey to examine the socio-economic gap in child malnutrition using a single anthropometric measure—CIAF—in India. More specifically, the study aimed to identify the maternal, child and household level factors responsible for the rich-poor gap in child malnutrition and quantify their contribution to this gap by using the multivariate decomposition technique.

## Methods

### Dataset and sample covered

This study used nationally representative data from the Comprehensive National Nutrition Survey (CNNS) in India, which adopted a multi-stage sampling design using probability proportional to size (PPS) sampling procedure, after geographical stratification of urban and rural areas, to select the primary sampling units. For smaller Primary sampling units (PSUs), the sampling design was conducted in two stages; in the first stage, PSUs were selected using PPS sampling and in the second stage, a systematic random selection of households was done within each PSU. In large PSUs, the sampling design involved three stages, with the addition of a segmentation procedure to reduce enumeration areas to manageable sizes. This cross-sectional household survey was designed to provide nationally representative and comprehensive nutritional profiling of preschoolers (0–4 years), children of school-going age (5–9 years) and adolescents (10–19 years). Children who had a chronic illness, physical deformity, mental illness, cognitive disability, or an ongoing current illness (high fever, infection) were not included in the study. Analyses for this study was conducted by utilizing anthropometric data of 38,060 children aged under 5 years and their biological mothers, along with other socio-economic indicators that were collected across all 30 states of India between February 24, 2016 and October 26, 2018. The procedure of arriving at a final analytical sample size is described in Table 1.

**Table 1:** Selection of analytical sample

Selection of analytical sample	N
Total sample collected	38,060
Biological mother is not the respondent	2608
Mothers’ education, age or schooling not known	132
Flagged and missing BMI of mother	3368
Flagged or missing anthropometric data of child	1452
**Analytical sample**	**30,500**

### Outcome variable

Weight was recorded in kilograms and height in centimeters, using a digital SECA scale and three-piece wooden height/length board, respectively. Before measurements were taken, the instrument was set up on a portable wooden square surface and a spirit level was used to ensure an even measurement surface. Recumbent length was measured in children who were either less than 2 years of age or 85 cm in length and for the remaining children, their standing height was measured [20]. WHO Anthro-Plus software was used to calculate children’s Z-scores standard deviation (SD) scores for height-for-age (HAZ), weight-for-age (WAZ) and weight-for-height (WHZ) [22–24]. Wasting was defined as weight-for-height (Z-Score [WHZ] < −2SD); stunting was defined as height-for-age (Z-Score [HAZ] < −2SD) and underweight was defined as weight-for-age (Z-Score [WAZ] < −2SD). CIAF subgroups are presented in Table 2. The sum of the children in groups B to Y provided the CIAF. Group A was excluded from the final estimates as children in this category had no anthropometric failure.

**Table 2:** Description of selected study variables

Variable	Description	Constructed variable
**Outcome Variable**		
Composite variable of anthropometric failure	Composite anthropometric failure calculated using all combinations of (HAZ, WHZ and HAZ)	0 = No Failure; 1 = Failure
**Covariates**		
Mother’s BMI (kg/m^2^)	Body Mass Index (Continuous, calculated using measured height and weight)	1 = Underweight (< 18.5 kg/m^2^); 2 = Normal (18.5–24.5 kg/m^2^); 3 = Overweight or Obese (> = 25.0 kg/m^2^)
Mother’s Education	Respondent educational level (0 = No education,1 = Educated)	Same variable used
Mother’s Employment Status	Respondent currently working (0 = Non-working, 1 = Working)	Same variable used
Mother’s Age	Current age of mother in completed years	1 = < 25 years; 2 = 25 years or more
Mother’s Mass Media Exposure	1. Frequency of reading newspaper or magazine 2. Frequency of listening to radio 3. Frequency of watching television (0 = Not at all, 1 = Occasionally, 2 = Atleast once a week, 3 = Daily)	0 = No (No Access); 1, 2 & 3 as 1 = Yes (Access to at least one of the information source)
Mother’s Parity	Total children ever born (Continuous)	1 = 1–2; 2 = 3 or more
Age of Child (in years)	Current age of child in completed years	1 = 0–2 year; 2 = 3–4 year
Sex of Child	Sex of child (1 = Male, 2 = Female)	Same variable used
Morbidity in past 2 weeks	1. Child suffered from diarrhoea in last 2 weeks; 2. Child suffered from acute respiratory infection in last 2 weeks; 3. Child suffered from fever in last 2 weeks (0 = No, 1 = Yes)	0 = No; 1, 2 & 3 as 1 = Yes (Suffered from atleast one ailment)
Wealth Index	Wealth index (1 = poorest, 2 = poorer, 3 = middle, 4 = richer, 5 = richest)	Same variable used
Place of Residence	Type of place of residence (1 = Rural, 2 = Urban)	Same variable used
Caste	1 = Scheduled Caste, 2 = Scheduled Tribe, 3 = Other Backward Caste (OBC), 4 = Others	1 = 1 & 2 (Scheduled caste/tribe); 2 = 3 & 4 (OBC/Others)

### Covariates

Based on the available literature [25–27], maternal, child and household characteristics associated with malnutrition were included in the analysis. Maternal covariates included age, education, employment status, mass media exposure and parity. Child covariates included age, sex, and morbidity in the past 2 weeks (diarrhea, respiratory infection, or fever). The wealth index was computed using data on the household’s ownership of specific assets, such as televisions and bicycles, materials used for housing construction, access to and type of water and sanitation facilities and the number and kind of other consumer goods they owned. The index was included as a household-level covariate. Households were given scores derived at using the principal component analysis adjusted for national and state-level weights. Wealth quintiles were computed by dividing the weighted distribution into five equal categories, each with 20% of the sample population. Other household-level covariates included caste and place of residence. The list of selected covariates and construction plan are provided in Table 2.

### Statistical analysis

Descriptive statistics were used to describe variables for the study. The association of CIAF with socio-economic characteristics was verified by Pearson’s χ2 test. Logistic regression was used to examine the association of CIAF with socio-demographic covariates. Results were presented as unadjusted odds ratios (UOR) and adjusted odds ratios (AOR) with 95% confidence intervals (CI). The estimates of CIAF prevalence in the population and all regression models were adjusted to consider the complex sampling design of the CNNS, 2016–18 by including primary sampling units, sampling weights and strata in the models.

To quantify the contribution of selected predictors in explaining the rich-poor gap in the prevalence of CIAF, multivariate decomposition analysis was used. Multivariate decomposition technique uses the output from regression models to partition the components of a group difference in a statistic, such as a mean or proportion, into a component attributable to compositional differences between groups, i.e., differences in characteristics or endowments, and a component attributable to differences in the effects of characteristics, i.e., differences in the returns, coefficients or behavioral responses. The mean difference in Y between groups A and B can be decomposed as,

$$ {Y}_A-{Y}_B=\overline{F\left({X}_A{\beta}_A\right)}-\overline{F\left({X}_B{\beta}_B\right)}=\underset{\mathrm{E}}{\underbrace{\left\{\overline{F\left({X}_A{\beta}_A\right)}-\overline{F\left({X}_B{\beta}_A\right)}\right\}}}+\underset{\mathrm{C}}{\underbrace{\left\{\overline{F\left({X}_B{\beta}_A\right)}-\overline{F\left({X}_B{\beta}_B\right)}\right\}}} $$

where Y denotes the N × 1 dependent variable vector, X is an N × K matrix of independent variables, and β is a K × 1 vector of the coefficient.

The component labeled E refers to the part of the differential attributable to differences in endowments or characteristics usually called the explained component or characteristics effects. C refers to the part of the differential attributable to differences in coefficients or effects usually known as the unexplained component or coefficient effects; where A is the richest children (comparison group) and B is the poorest children (reference group). Therefore, E reflects a counterfactual comparison of the difference in outcomes from the richest children’s perspective and C reflects a counterfactual comparison of outcomes from the poorest children’s perspective. STATA 16.0 software was used for data analysis.

### Ethical review

The CNNS received ethical clearance from the Ethical Review Board of the Post Graduate Institute for Medical Education and Research (PGIMER) and the Institutional Review Board of the Population Council in New York. All aspects of the survey were informed, following which written consent was obtained from caregivers of children aged under 5 years.

## Results

The findings of this study show an overall CIAF prevalence of 48.2% among children aged under 5 years. 19.1% of children suffered from only one form of anthropometric failure (groups B, F and G), which include wasting only (4.6%), stunting only (11.5%) and underweight only (3.0%). 6.5% of the children suffered from both wasting & underweight (group C) and 16.6% were stunted as well as underweight (group E), constituting 22.1% of children with two simultaneous failure groups (group C and E). As shown in Table 3, 6.0% of children had all three forms of anthropometric failures. The distribution of covariates among poorest and richest socio-economic groups is presented in supplementary Table 1.

**Table 3:** Classification of anthropometric failure as per CIAF and distribution of participants as per CIAF categories^a^

Categories of Undernutrition		Wasting	Stunting	Underweight	***N*** = 30,500	(%)
**A**	No failure	No	No	No	18,434	51.8
**B**	Wasting (Low weight-for-height WTHT) only	Yes	No	No	1392	4.6
**C**	Wasting and Underweight [Low weight-for-height (WTHT) and low weight-for-age (WTA)]	Yes	No	Yes	1729	6.5
**D**	Wasting, Stunting and Underweight (All three anthropometric failures)	Yes	Yes	Yes	1222	6.0
**E**	Stunting and Underweight [Low height-for-age (HTA) and Low weight-for-age (WTA)]	No	Yes	Yes	3552	16.6
**F**	Stunting only [Low height-for-age (HTA)]	No	Yes	No	3467	11.5
**G**	Underweight only [Low weight-for-age (WTA)]	No	No	Yes	704	3.0
**Overall CIAF**	**B + C + D+ E + F + G**				**12,066**	**48.2**

^a^ Another theoretical combination would be the children who were “wasted and stunted” but not underweight. This combination is not physically possible since a child cannot have a low WTHT and low HTA and not be underweight

Table 4 presents the percentage of children under the age of 5 years who had anthropometric failure based on maternal, birth and socio-demographic characteristics. CIAF was found in 63.1% children from poorest households, whereas only 31.5% children from richest household experienced CIAF. 50.5% children from rural areas and those with morbidity in the 2 weeks prior to survey had CIAF. CIAF was found in 59.1% and 32.1% of children with underweight and overweight/obese mother respectively and in 60% of children with mothers who had no formal schooling, 46% whose mothers were not working and more than half with mothers who were not exposed to mass media.

**Table 4:** Percentage of the children under-five who had anthropometric failure by their maternal, birth and socio-demographic characteristics

Covariates	Composite indicator of anthropometric failure (CIAF)		Total	
	% (95% CI)	***p***-value	n	% (95% CI)
**Household Characteristics**				
**Wealth Index**		< 0.001		
Poorest	63.1 (60.2–65.8)		2391	19.6 (17.3–22.0)
Poor	55.3 (51.8–58.7)		3613	20.1 (18.7–21.5)
Middle	49.5 (47.2–51.8)		5734	19.9 (18.7–21.1)
Rich	42.4 (40.3–44.4)		7931	20.0 (18.8–21.3)
Richest	31.5 (29.7–33.4)		10,831	20.5 (18.9–22.2)
**Place of Residence**		< 0.001		
Rural	50.5 (48.9–52.2)		16,542	76.4 (73.8–78.9)
Urban	40.6 (38.9–42.4)		13,958	23.6 (21.1–26.2)
**Caste**		< 0.001		
Scheduled Caste/Tribe	52.7 (50.9–54.6)		11,347	35.6 (33.3–38.1)
Others/OBC	45.7 (44.0–47.4)		19,153	64.4 (61.9–66.7)
**Maternal Covariates**				
**Mother’s BMI**		< 0.001		
Underweight	59.1 (56.8–61.3)		5683	28.1 (26.8–29.4)
Normal	47.2 (45.4–48.9)		17,662	56.6 (55.4–57.7)
Overweight/obese	32.1 (30.1–34.3)		7155	15.4 (14.3–16.5)
**Mother’s Education**		< 0.001		
No education	60.1 (57.8–62.3)		5617	30.4 (28.2–32.6)
Educated	43.0 (41.6–44.4)		24,883	69.6 (67.4–71.8)
**Mother’s Employment Status**		< 0.001		
Not working	46.0 (44.5–47.6)		23,274	76.0 (74.3–77.6)
Working	55.1 (53.0–57.2)		7226	24.0 (22.4–25.7)
**Mother’s Age**		< 0.001		
< 25 years	47.9 (46.0–49.9)		8442	33.3 (32.1–34.6)
> =25 years	48.3 (46.8–49.9)		22,058	66.6 (65.4–67.8)
**Mother’s Access to Information**		< 0.001		
No	57.5 (55.5–59.5)		6980	39.7 (37.1–42.4)
Yes	42.1 (40.6–43.6)		23,520	60.3 (57.6–62.9)
**Mother’s Parity**		< 0.001		
1–2	45.8 (44.3–47.2)		23,847	72.3 (70.8–73.7)
3 or more	54.6 (52.5–56.6)		6653	27.7 (26.3–29.2)
**Child Covariates**				
**Age (in years)**		0.021		
0–2	47.8 (46.1–49.4)		17,943	59.5 (58.3–60.6)
2–4	48.8 (46.8–50.9)		12,557	40.5 (39.4–41.7)
**Sex**		0.011		
Male	48.5 (46.7–50.2)		16,062	52.0 (50.8–53.2)
Female	47.9 (46.1–49.7)		14,438	48.0 (46.8–49.2)
**Morbidity in Past 2 Weeks**		< 0.001		
No	46.0 (44.4–47.6)		17,385	50.7 (49.2–52.2)
Yes	50.5 (48.6–52.4)		13,115	49.3 (47.8–50.8)

Table 5 presents the factors associated with CIAF. In the multivariate analysis of socio-demographic factors, the odds of CIAF were more likely among children belonging to poorest households (AOR: 2.41, 95% CI: 2.12–2.75), Scheduled Caste/Scheduled Tribes (SC/ST) households (AOR: 1.06, 95% CI: 1.01–1.12) and those residing in urban area (AOR: 1.06, 95% CI 1.00–1.11). Children of mothers who were underweight and had high parity were at higher risk of CIAF (AOR: 1.51, 95% CI: 1.42–1.61 and AOR: 1.15, 95% CI: 1.08–1.22) respectively. Children with mothers who were exposed to mass media were at a lower risk of CIAF (AOR: 0.87, 95% CI: 0.81–0.93). Elder children in age group 2–4 years (AOR: 1.07, 95% CI: 1.02–1.12), male children (AOR: 1.07, 95% CI: 1.02–1.12) and those with morbidity in the 2 weeks prior to survey (AOR: 1.07, 95% CI: 1.02–1.13) were at a higher risk of CIAF.

**Table 5:** Association between socio-demographic characteristics with CIAF among infants in India, CNNS 2016–18

Covariates	Composite indicator of anthropometric failure (CIAF)	
	UOR^**a**^	AOR^**b**^
**Household Characteristics**		
**Wealth Index**		
Poorest	4.31 (3.92–4.72)***	2.41 (2.12–2.75)***
Poor	3.01 (2.78–3.25)***	2.00 (1.81–2.22)***
Middle	2.18 (2.04–2.33)***	1.65 (1.53–1.79)***
Rich	1.62 (1.53–1.73)***	1.42 (1.33–1.52)***
Richest	Ref	Ref
**Place of Residence**		
Urban	Ref	Ref
Rural	0.69 (0.66–0.72)***	1.06 (1.00–1.11)*
**Caste**		
SC/ST	1.29 (1.23–1.36)***	1.06 (1.01–1.12)**
General/OBC	Ref	Ref
**Maternal Covariates**		
**Mother’s BMI**		
Underweight	1.76 (1.65–1.87)***	1.51 (1.42–1.61)***
Normal	Ref	Ref
Overweight/obese	0.56 (0.53–0.60)***	0.7 (0.65–0.74)***
**Mother’s Education**		
Uneducated	2.09 (1.97–2.21)***	1.26 (1.18–1.35)***
Educated	Ref	Ref
**Mother’s Employment Status**		
Non-working	Ref	Ref
Working	1.28 (1.21–1.35)***	1.05 (0.99–1.11)*
**Mother’s Age**		
< 25 years	1.16 (1.1–1.22)***	1.04 (0.98–1.1)
> =25 years	Ref	Ref
**Mother’s Access to Information**		
No	Ref	Ref
Yes	0.48 (0.46–0.51)***	0.87 (0.81–0.93)***
**Mother’s Parity**		
1–2	Ref	Ref
3 or more	1.52 (1.44–1.60)***	1.15 (1.08–1.22)***
**Child Covariates**		
**Age (in years)**		
0–2	Ref	Ref
2–4	1.06 (1.01–1.11)**	1.07 (1.02–1.12)**
**Sex of Child**		
Male	1.06 (1.01–1.11)**	1.07 (1.02–1.12)**
Female	Ref	Ref
**Morbidity in Past 2 Weeks**		
No	Ref	Ref
Yes	1.13 (1.08–1.18)***	1.07 (1.02–1.13)***

^a^Unadjusted odds ratio; ^b^Adjusted odds ratio

*** p < 0.01, ** p < 0.05, * p < 0.1

Table 6 shows the how the endowment and coefficient effects contribute to the gap in CIAF prevalence between children of poorest and richest households. A negative contribution indicates that the determinant narrowed the gap between poorest and richest households and vice-versa. Results showed that the differences due to coefficient accounted for 59.45% of the observed socio-economic differential in the prevalence of CIAF and the difference due to characteristics accounted for 40.55%. For instance, if the mothers from the poorest households were as educated to those from the richest households, then the prevalence of CIAF would reduce by 15.03%. Similarly, equalizing parity of mothers and their exposure to mass media in poorest and richest households could reduce the prevalence of CIAF by 5.38 and 4.91%, respectively. The difference in mothers’ body mass index (BMI) among the poorest and richest households contributed to a gap of 13.6%—underweight: 6.47% and overweight/obese: 7.13%)—in the prevalence of CIAF.

**Table 6:** Decomposition of difference in composite indicator of anthropometric failure (CIAF) between the richest and the poorest socioeconomic groups

Variable	Difference due to characteristics (Explained)			Difference due to coefficients (Unexplained)		
	Coefficient	Percent	***p***-value	Coefficient	Percent	***p***-value
Mother’s education	−0.05	15.03	0.002	−0.02	6.00	0.092
Mother’s exposure to mass media	− 0.02	4.91	0.433	0.01	−3.57	0.071
Mother’s parity	−0.02	5.38	< 0.001	0.07	−21.40	0.058
Underweight mother (as per BMI)	−0.02	6.47	< 0.001	0.00	1.39	0.595
Mother with normal weight (as per BMI)	0.00	−0.04	0.676	0.01	−1.82	0.602
Overweight/obese mother (as per BMI)	−0.02	7.13	< 0.001	0.00	−0.01	0.964
Child’s age	0.00	−0.04	0.565	0.00	0.27	0.978
Child’s sex	0.00	−0.02	0.010	−0.06	18.09	0.065
Child’s morbidity	−0.00	0.28	0.082	0.00	1.12	0.712
Place of residence	0.00	−0.91	0.599	−0.07	21.37	0.068
Caste	−0.01	2.35	0.018	−0.06	17.79	0.073
**Constant**				**−0.07**	**20.20**	**0.457**
**Total**	**−0.14**	**40.55**	**< 0.001**	**−0.21**	**59.45**	**< 0.001**

## Discussion

This study attempted to estimate the overall burden of child undernutrition at the national level by calculating CIAF and the factors contributing to the rich-poor gap in the prevalence of CIAF. The overall prevalence of CIAF was reported as 48.1%, which was in line with the estimates reported by studies conducted in Assam and Bangladesh [28, 29]. However, studies conducted in states of Odisha, West Bengal and Chhattisgarh reported higher prevalence of CIAF [30–32]. Similarly, studies conducted in Ethiopia [33] and Nepal [31] reported higher prevalence, whereas studies from Zimbabwe and Peru reported lower prevalence of CIAF [34]. The findings of this study align with the findings of previous studies [26–29] and show that stunting is the most prevalent type of undernutrition. These findings suggest that 16.6% of children experienced both stunting and underweight, indicating the coexistence of both acute and chronic undernutrition and emphasizing the importance of a composite index to gauge the scale of undernutrition.

Several socio-demographic, maternal and child related factors were found to be associated with the prevalence of CIAF in this study. Children belonging to poor households were found to be at a higher risk of being undernourished as compared to children from rich households. Systematic review conducted in low- and middle-income countries, including India, reported that percent point difference between the poorest and richest households remains the same overtime [35]. This study further revealed that maternal nutrition, education, parity, and exposure to mass media were associated with child undernutrition and subsequently with the overall CIAF.

Maternal undernutrition in form of low stature or underweight is consequent to prevailing adverse social and economic circumstances. Mothers who are undernourished in their formative years are more likely to deliver small-for-gestational age (SGA) babies, who then are at a higher risk of undernutrition [36]. Findings of this study suggest that children of a mothers with no or lower education, no exposure to mass media and high parity were associated with CIAF. These results corroborate findings from other studies that illustrate how low education and no exposure to mass media limit a mother’s knowledge and consequently result in poor awareness for nutritional programs and child feeding practices, leading to child undernutrition [37, 38].

Male children in this study were found at a greater risk of being undernourished as per CIAF. This finding was contrary to other studies conducted in developing countries with the standalone undernutrition indicators [39]. However, a meta-analysis conducted in sub-Saharan Africa reported that more male children were stunted than females, suggesting males as more vulnerable to health inequalities than females [40]. Further research is needed to understand this anomaly. Findings of this study suggest that an increase in the child’s age was positively associated with undernutrition. These findings are in line with previous studies [41, 42]. One probable reason for this could be that a child’s nutritional needs are not fulfilled as per the demand with increasing age.

Another objective of this study was to disaggregate the effect of the determinants in explaining the gap in prevalence of CIAF between rich and poor households in India. The results revealed that 41% of the gap in prevalence of CIAF was attributed mainly to the distribution of determinants between poor and rich households. Maternal factors were found as main contributors to endowment effect, explaining most of the gap in the prevalence of CIAF. Low level of maternal education in poor households limit the practice of healthy behavior for reasons such as limited knowledge, poor nutritional practices, lack of resources, underutilization of health care services and depleted autonomy within the household. These limitations to the practice of healthy behavior are detrimental factors that affect child nutritional outcome [38]. The nutritional status of mothers among poor household is another important factor responsible for the poor nutritional status of children. Intrauterine growth retardation in malnourished mothers is known to be directly associated with premature deliveries and low birth-weight infants, leading to different morbidities along with undernutrition in early childhood [43]. Mother’s exposure to mass media plays a vital role in reducing the rich poor gap. Mothers with exposure to mass media are better informed on breastfeeding practices, government health initiatives and other programs that promote the health of children [44]. Caste was another contributor to the endowment effect. The significance of this may be attributed to the fact that deprived caste groups such as the SC/ST are clustered primarily in unhealthy living environments as compared to the remaining population. In contrast, other caste groups are characterized by a relatively better socioeconomic status and are thus at a lower risk of childhood undernutrition [45].

One of the limitations in this study is non-availability of household level factors related to water, sanitation and hygiene and maternal nutrition like anemia and other factors like maternal autonomy, which might influence the status of child nutritional, have not been assessed in this study. Further research is needed to explore the role of these variables in anthropometric failure among under-five children.

With the launch of *Poshan Abhiyaan*, India is striving to achieve SDG-2, that aims to end all forms of malnutrition by 2030 [46]. While the importance of conventional standalone indicators to screen undernutrition is well documented, screening through them may lead to exclusion of those who demonstrate multiple manifestations. In addition, information from conventional indicators in the overall change in undernutrition is contradictory at times. The CIAF takes the differences between the three conventional indicators into account and provide an indicator that can be used to identify nutritionally vulnerable geographies as well as segments of the population. Findings from this study can be helpful to identify groups for targeted interventions that aim to reduce rich-poor inequity.

## Conclusion

There were two key findings of this study. Firstly, a composite index to assess the overall anthropometric failure is essential as it provides a broader understanding of the extent and pattern of undernutrition among children. Secondly, findings suggest that intervention programs with a targeted approach are needed to reach the most vulnerable groups and should devise policy to strengthen the factors which will narrow the rich-poor gap and further reduce the overall burden of undernutrition.

## Supplementary Information

**Additional file 1: Supplementary Table 1.** Percentage of the children under-five by their maternal, birth and socio-demographic characteristics between the richest and the poorest socioeconomic groups.

## Data Availability

Reasonable request for data used in this article may be made to the corresponding author.
