# Supplementary material for: Socio-economic inequality in anthropometric failure among children aged under 5 years in India: evidence from the Comprehensive National Nutrition Survey 2016–18
Source: Int J Equity Health. 2021 Jul 30;20:176. doi: 10.1186/s12939-021-01512-4 (PMC8325297; doi:10.1186/s12939-021-01512-4)
Supplement: Supplementary file 1 — Additional file 1: Supplementary Table 1. Percentage of the children under-five by their maternal, birth and socio-demographic characteristics between the richest and the poorest socioeconomic groups. [file 12939_2021_1512_MOESM1_ESM.docx]

| **Supplementary Table 1: Percentage of the children under-five by their maternal, birth and socio-demographic characteristics between the richest and the poorest socioeconomic groups** | | |
| --- | --- | --- |
| **Covariates** | **Poorest (N=2391)** | **Richest (N=10831)** |
|  | **%** | **%** |
| **Household Characteristics** |  |  |
| **Place of Residence** |  |  |
| Rural | 89.7 | 32.2 |
| Urban | 10.3 | 67.8 |
| **Caste** |  |  |
| Scheduled Caste/Tribe | 56.5 | 25.3 |
| Others/OBC | 43.5 | 74.7 |
| **Maternal Covariates** |  |  |
| **Mother's BMI** |  |  |
| Underweight | 38.6 | 9.2 |
| Normal | 57.4 | 53.1 |
| Overweight/obese | 4.0 | 37.7 |
| **Mother's Education** |  |  |
| No education | 65.5 | 3.2 |
| Educated | 34.6 | 96.8 |
| **Mother's Employment Status** |  |  |
| Not working | 56.5 | 82.0 |
| Working | 43.5 | 18.0 |
| **Mother's Age** |  |  |
| <25 years | 27.5 | 19.1 |
| >=25 years | 72.5 | 80.9 |
| **Mother's Access to Information** |  |  |
| No | 82.7 | 3.1 |
| Yes | 17.3 | 96.9 |
| **Mother's Parity** |  |  |
| 1-2 | 52.7 | 88.5 |
| 3 or more | 47.3 | 11.5 |
| **Child Covariates** |  |  |
| **Age (in years)** |  |  |
| 0-2 | 57.2 | 59.6 |
| 2-4 | 42.8 | 40.5 |
| **Sex** |  |  |
| Male | 52.9 | 53.2 |
| Female | 47.1 | 46.8 |
| **Morbidity in Past 2 Weeks** |  |  |
| No | 54.8 | 60.5 |
| Yes | 45.2 | 39.5 |
